# Enduring glucocorticoid-evoked exacerbation of synaptic plasticity disruption in male rats modelling early Alzheimer’s disease amyloidosis

**DOI:** 10.1038/s41386-021-01056-9

**Published:** 2021-06-29

**Authors:** Yingjie Qi, Igor Klyubin, Tomas Ondrejcak, Neng-Wei Hu, Michael J. Rowan

**Affiliations:** 1 Department of Pharmacology & Therapeutics and Institute of Neuroscience, Trinity College, Dublin, 2 Ireland; 2 Department of Physiology and Neurobiology, School of Basic Medical Sciences, Zhengzhou University, 100 Science Avenue, Zhengzhou, 450001 China

**Keywords:** Alzheimer's disease, Long-term potentiation, Hippocampus

## Abstract

Synaptic dysfunction is a likely proximate cause of subtle cognitive impairment in early Alzheimer’s disease. Soluble oligomers are the most synaptotoxic forms of amyloid ß-protein (Aß) and mediate synaptic plasticity disruption in Alzheimer’s disease amyloidosis. Because the presence and extent of cortisol excess in prodromal Alzheimer’s disease predicts the onset of cognitive symptoms we hypothesised that corticosteroids would exacerbate the inhibition of hippocampal synaptic long-term potentiation in a rat model of Alzheimer’s disease amyloidosis. In a longitudinal experimental design using freely behaving pre-plaque McGill-R-Thy1-APP male rats, three injections of corticosterone or the glucocorticoid methylprednisolone profoundly disrupted long-term potentiation induced by strong conditioning stimulation for at least 2 months. The same treatments had a transient or no detectible detrimental effect on synaptic plasticity in wild-type littermates. Moreover, corticosterone-mediated cognitive dysfunction, as assessed in a novel object recognition test, was more persistent in the transgenic animals. Evidence for the involvement of pro-inflammatory mechanisms was provided by the ability of the selective the NOD-leucine rich repeat and pyrin containing protein 3 (NLRP3) inflammasome inhibitor Mcc950 to reverse the synaptic plasticity deficit in corticosterone-treated transgenic animals. The marked prolongation of the synaptic plasticity disrupting effects of brief corticosteroid excess substantiates a causal role for hypothalamic-pituitary-adrenal axis dysregulation in early Alzheimer’s disease.

## Introduction

Because Alzheimer’s disease is predominantly sporadic many non-genetic, potentially modifiable, factors have been posited to play a key role in its pathogenesis. In particular, aberrant activation of the hypothalamic–pituitary–adrenal axis is considered to make a significant contribution, even at the early stages [1–4]. Indeed, the extent of cortisol excess provides a means to accurately predict the onset of clinical symptoms [5]. In most Alzheimer’s disease cases it is difficult to determine what is driving the abnormal endocrine state, although it is generally assumed to be caused by excess psychological or cellular stress [4]. For example, Alzheimer’s disease patients often display symptoms of major depressive disorder, a disease which doubles the risk for Alzheimer’s disease [6] and it seems likely that Alzheimer’s disease and depression lie on a continuum, sharing common glucocorticoid-mediated mechanisms [7]. Furthermore, the widespread clinical use of glucocorticoids is well known to cause mood and cognitive adverse effects [8, 9].

Exogenous administration of stress levels of corticosteroids over several weeks is used to model depressive syndromes in adult rodents [10]. Apart from emotional symptoms, such long-term exposure triggers age-related cognitive problems [10–12]. Synaptic pathology in brain areas such as the hippocampus is a likely proximate cause of impairment of learning and memory, with connections within and between certain networks being particularly vulnerable [13–15]. Of particular note, tau pathogenic mechanisms have been strongly implicated in mediating such synaptic and cognitive impairment [7].

In addition to triggering synaptic and tau pathology chronic administration of glucocorticoids exacerbates amyloid pathology, with accelerated amyloid deposition and cognitive deficits in transgenic mice overexpressing amyloid precursor protein (APP) with familial Alzheimer’s disease mutations [16]. Indeed blocking glucocorticoid receptors can not only abrogate memory and synaptic deficits but also reduces amyloid load in APP transgenic mice [16–19]. Soluble amyloid ß (Aß) oligomers are particularly implicated in mediating disruption of synaptic plasticity [20] and recently the acute inhibition of long-term potentiation (LTP) by Aß in hippocampal slices was reported to depend on glucocorticoid receptor activation [21].

Although Aß-induced synaptic deficits are regulated by glucocorticoid status, and prolonged corticosteroid exposure can exacerbate amyloidosis-related synaptic “loss”, it is not known if, and for how long, synaptic function in the pre-plaque stage is more susceptible to disruption by corticosteroid elevation. Here, we assess if even relatively brief elevations in corticosterone (CORT), the rodent equivalent of cortisol, exacerbate hippocampal synaptic plasticity dysfunction in young APP transgenic rats, prior to amyloid plaque deposition. These animals have an age-dependent Aß oligomer-mediated inhibition of LTP which necessitates microglial NOD-leucine rich repeat and pyrin containing protein 3 (NLRP3) inflammasome activation [22, 23]. Since CORT can also activate this pro-inflammatory mechanism in the hippocampus [24], we hypothesised that inhibition of the inflammasome would reverse any persistent synaptic dysfunction.

## Materials and methods

### Animals

Male transgenic rats (4–5 months old at start) expressing human APP751 with Swedish and Indiana mutations under the control of the murine Thy1.2 promoter (McGill-R-Thy1-APP) [25] and their wild-type littermates were genotyped commercially by Transnetyx (Cordova, TN, USA) using real time polymerase chain reaction. McGill-R-Thy1-APP rats have an age-dependent accumulation of Aβ, first intracellularly, and then in extracellular neuritic plaques. Cognitive function is impaired before the formation of plaques, as early as 3 months of age [25]. We chose to inject animals at an age (4–5 month) when LTP is impaired by the build-up of Aß oligomers but prior to the deposition of plaques [26, 27]. All experiments were carried out in accordance with the approval of the Health Products Regulatory Authority, Ireland, using methods similar to those described previously [22]. Animals had free access to food and water and a 12-h lights on/off cycle.

### In vivo surgery and electrophysiology

For the chronic recording in the longitudinal studies the implantation procedure was carried out under anaesthesia using a mixture of ketamine and xylazine (80 and 8 mg/kg, respectively, i.p.) according to methods similar to those described previously [22]. For the longitudinal recovery experiments the rats were allowed at least 14 days after surgery before recordings began [22]. These rats were housed individually in their home cages post-surgery between recording sessions.

Both recording and stimulating electrodes were constructed from Teflon coated tungsten wires. The recording electrodes were single-strand wires (75 μm inner core diameter, 112 μm external diameter) targeted at 3.8 mm posterior to bregma and 2.5 mm lateral to midline, whereas the stimulating electrodes were twisted-pair wires (75 μm inner core diameter, 112 μm external diameter) targeted at 4.6 mm posterior to bregma and 3.8 mm lateral to midline. Field excitatory postsynaptic potentials (EPSPs) were recorded from the stratum radiatum in the CA1 area of the dorsal hippocampus in response to stimulation of the ipsilateral Schaffer collateral/commissural pathway. The final placement of electrodes was optimised by using electrophysiological criteria and confirmed via post-mortem analysis [22].

Test stimuli were delivered to the Schaffer-collateral/commissural pathway every 30 s to evoke field EPSPs that were 45–60% maximum amplitude. To induce LTP we used a strong high frequency stimulation (sHFS) protocol consisting of 3 sets of 10 trains of 20 stimuli at high intensity (75% maximum) at 400 Hz with an inter-train interval of 2 s and an inter-set interval of 5 min [22]. The form of LTP induced by sHFS is both NMDA receptor-dependent and voltage-gated Ca2+ channel-dependent [26, 28]. For non-recovery experiments, only used in the Mcc950 study, the implantation procedure was comparable [22] but the rats were anaesthetised with urethane (1.5 g/kg, i.p.). Core body temperature was maintained at 37.5 ± 0.5 °C.

Recovery animal experiments were carried out in a well-lit room. The recording compartment consisted of the base of the home cage, including normal bedding and food/water, but the sides were replaced with a translucent Perspex plastic box (27 × 22 × 30 cm) with an open roof. The rats had access to food and water throughout the whole recording session from the same position as in the home cage. All animals were first habituated to the recording procedure over the post-surgery recovery period [22].

### Behavioural tasks

Each animal was handled for 5 min per day for a week prior to testing. For the open field the rat was placed in the centre of an open square white apparatus (L × W × H, 60 × 60 × 40 cm) and allowed to explore it for 5 min. Two parameters were measured: number of lines (marked on the floor) crossed and time spent in the central area (35 × 35 cm). Twenty four hour after the open field, novel object recognition (NOR) test was performed in the same apparatus, using previously described methods [29]. During the sample phase two identical objects were presented to the animal for 5 min. In the retention phase 3 h later, one of the familiar objects was replaced with a novel one and the animal was allowed to explore them for a further 5 min. The NOR task was repeated again in either 14 or 20 days using another set of objects completely novel to the animals. The discrimination index was calculated as the duration of novel object interaction/ duration of total interaction with both objects.

### Drugs

CORT, Sigma was administered using polyethylene glycol 400 (PEG, Sigma, 1 ml/kg s.c.) as the vehicle. We chose a repeated dosing schedule over 3 days based on both previously published data reporting that 20 mg/kg is the minimum chronic dose necessary to trigger ‘depression-like’ symptoms in wild-type rats [10] and pilot experiments in which we found that 2 h after a single injection of CORT (10 mg/kg s.c.) LTP appeared normal in all animals tested regardless of genotype (WT, 156 ± 9%, *n* = 5 and TG, 152 ± 12%, *n* = 6). Both 6α-methylprednisolone 21-hemisuccinate sodium salt (MPL, Sigma) and Mcc950 sodium salt [30] (also known as CRID3; MedChemtronica, Sweden) were dissolved in H_2_O vehicle (1 ml/kg s.c. and i.p., respectively). Although by no means receptor specific, MPL has been reported to be at least 100-fold more active than CORT at glucocorticoid over mineralocorticoid receptors in vitro [31, 32]. Based on published work reporting the pharmacokinetics/pharmacodynamics of MPL [33–35], we chose a dose of 50 mg/kg. Previously, a sub-chronic dose 30 mg/kg was found not to affect NMDA receptor-dependent LTP in wild-type rats [36]. Choice of the s.c., as opposed to the oral or i.p., route of administration for CORT and MPL avoids first pass metabolism [37] and promotes a gradual increase in concentration in the systemic circulation and hence the brain [38]. The dose schedule for Mcc950 was based on one found to reverse the impairment of NMDA receptor-dependent LTP in McGill-R-Thy1-APP rats [23].

### Statistical analysis

Synaptic plasticity varies greatly depending on the stage of oestrous cycle (e.g. [39, 40]). Consistent with this literature, in pilot experiments we found that hippocampal LTP induced by either 200 or 400 Hz protocols was extremely variable in both wild type and transgenic female rats. In order to minimise animal numbers we restricted the present study to males. Animal numbers were also reduced by using a longitudinal design in the majority of experiments, requiring each animal to be recorded from multiple times and a repeated measures statistical evaluation.

The strength of synaptic transmission is expressed as a percentage of the EPSP amplitude recorded over a 30-60 min baseline recording period. The magnitude of LTP was measured at 3 h post-HFS and expressed as the mean ± SEM % baseline. No data were excluded. Control experiments were interleaved randomly throughout experimental sets. The choice of sample size was based on a priori testing to ensure adequate statistical power comparable to previously published papers (https://clincalc.com/stats/samplesize.aspx). Experimental data passed Shapiro-Wilk and Kolmogorov–Smirnov normality tests. For statistical analysis and graphical display EPSP amplitude measurements were grouped into 10 min epochs. In longitudinal studies one-way ANOVA with repeated measures, followed by Bonferroni’s multiple comparison test, was used to compare multiple time points in the same animals. In the cross-sectional Mcc950 study, one-way ANOVA, again followed by Bonferroni’s multiple comparison test, was used to compare the magnitude of LTP between multiple groups. Paired and unpaired Student’s *t* tests were used to compare between two time points within one group and between two groups, respectively. One-sample *t* test was used to analyse the bias towards the novel object [41]. A *p* value of <0.05 was considered statistically significant. Additional statistical information is available in Supplementary Tables 1 and 2.

## Results

### Repeated strong conditioning stimulation reliably triggers robust LTP in freely behaving McGill-R-Thy1-APP transgenic rats and their wild-type littermates

Previously, we reported that LTP induced by 200 Hz, but not sHFS repeated high intensity 400 Hz trains, is inhibited in an age-dependent manner in pre-plaque transgenic rats [22]. In this study we confirmed that there was no difference in the magnitude of sHFS-induced LTP between genotypes at 4–5 months of age (WT 161 ± 5% pre-sHFS baseline EPSP amplitude, *n* = 24 vs. TG 153 ± 4%, *n* = 26, *p* > 0.05, unpaired *t* test, data not shown). Therefore, we utilised the strong conditioning protocol to evaluate the effects of CORT in both wild-type and transgenic rats. Recordings were taken repeatedly in freely behaving chronically implanted animals. Because we used a longitudinal experimental design it was necessary to assess if the application of sHFS triggered similar magnitude LTP over the 2.5 month study period. Indeed, repeated application of the same strong protocol induced similar magnitude LTP at all recording sessions in both wild-type (157 ± 9% for session 0 month and 157 ± 12% for session 2.5 month) and transgenic (153 ± 6% for session 0 month and 153 ± 8% for session 2.5 month) animals (Supplementary Fig. 1).

### Sub-chronic CORT-evoked transient inhibition of LTP in wild-type rats

**Fig. 1:**
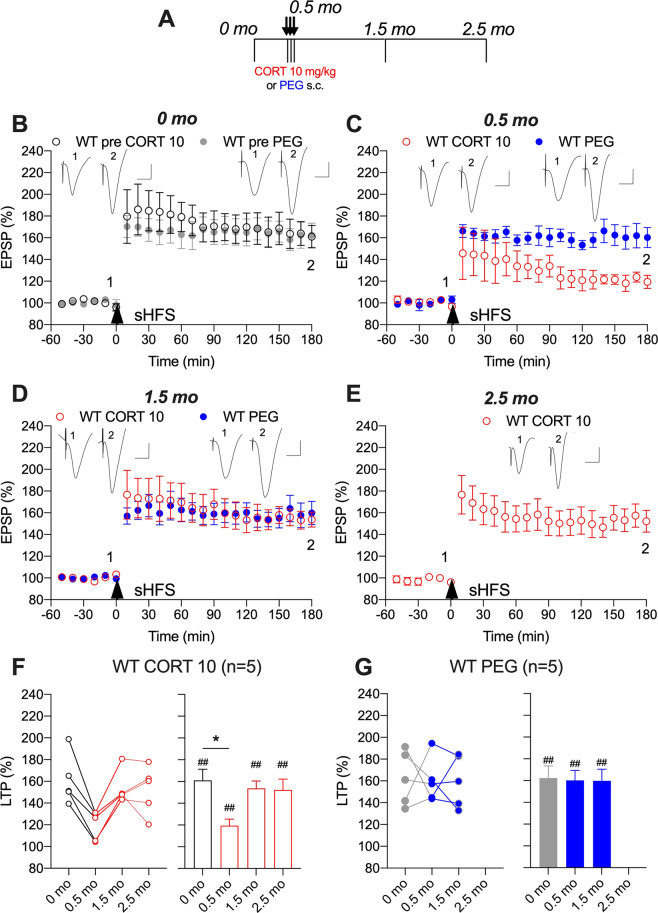
Corticosterone transiently disrupts synaptic plasticity in wild-type rats. **A** In a longitudinal study design, wild-type (WT) rats received single daily s.c. injections of either CORT (10 mg/kg, open circles) or vehicle (PEG, closed circles) for 3 days. Strong high frequency conditioning stimulation (sHFS, arrowhead) was applied repeatedly in the hippocampus of freely behaving rats over a 2.5 month period. The time course of sHFS-induced potentiation from the same animals is displayed before (**B**, 0 month), 2 h after (**C**, 0.5 month), 1 (**D**, 1.5 month) and 2 (**E**, 2.5 month) months after the last injection, starting 4 months of age. Red and blue colours indicate CORT 10 or PEG treatments, respectively (online version only). Insets show representative field EPSP traces at the times indicated. Calibration bars: vertical, 1 mV; horizontal, 10 ms. The magnitude of potentiation 3 h post-sHFS (last 10 min) at the different recording sessions is plotted for WT CORT 10 (**F**) and WT PEG (**G**) rats, for individuals (left hand panel) and groups (right hand panel). The ♯ symbol stands for a statistical comparison between pre- and 3 h post-HFS values at each recording session within one group (paired *t* test) whereas an * indicates a comparison between groups. The magnitude of LTP was not significantly different between recording sessions in WT PEG group (*p* > 0.05; one-way ANOVA with repeated measures). In contrast, LTP was significantly inhibited at 0.5 month in WT CORT 10 group. One symbol, *p* < 0.05; two symbols, *p* < 0.01. Values are mean ± S.E.M. % pre-HFS baseline EPSP amplitude. Additional statistical information for this and the other figures is available in Supplementary Table 1.

We first examined the effect of CORT on sHFS-induced LTP in wild-type rats. Sub-chronic treatment (10 mg/kg s.c. daily for 3 days) impaired LTP induced 2 h after the last injection (119 ± 6%, *p* < 0.05 compared with 160 ± 10% of pre-treatment LTP, one-way ANOVA with repeated measures, Fig. 1B, C, F). The later phase of LTP appeared especially vulnerable to disruption. Importantly, the impairment of LTP by sub-chronic CORT in wild-type rats was transient. Thus, LTP was no longer inhibited when tested in the same animals 1 month after the last injection of CORT (152 ± 10%, *p* > 0.05 compared with pre-treatment LTP, Fig. 1D, F). By contrast, similar magnitude LTP was triggered at all time points in vehicle-treated wild-type rats (Fig. 1).

### Sub-chronic CORT-evoked persistent inhibition of LTP in APP transgenic rats

**Fig. 2:**
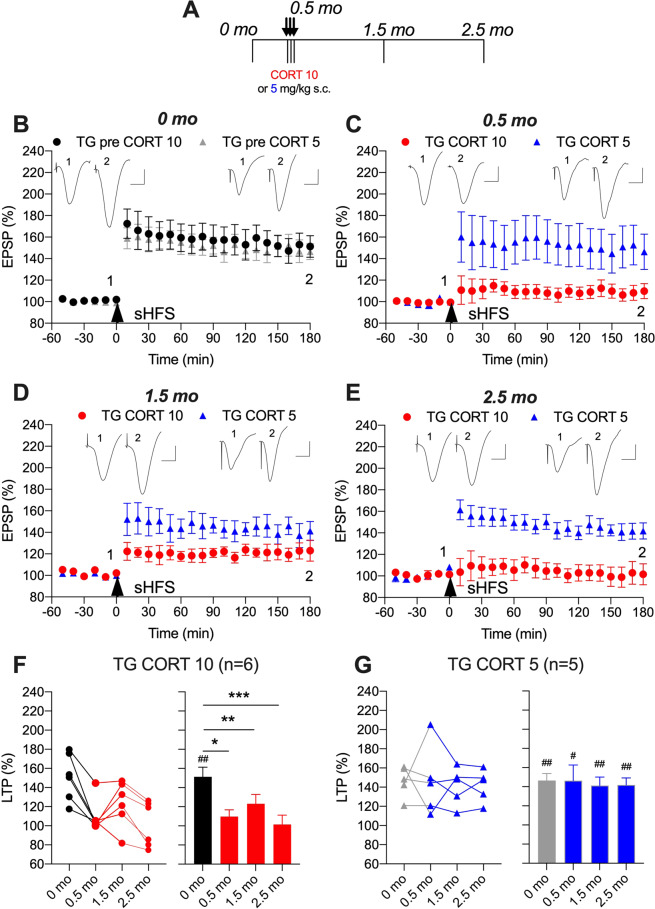
Corticosterone persistently disrupts synaptic plasticity in APP transgenic rats. **A** In a longitudinal study design, transgenic (TG) rats received single daily s.c. injections of CORT (either 10 mg/kg, closed circles, or 5 mg/kg, open circles) for 3 days. Strong high frequency conditioning stimulation (sHFS, arrowhead) was applied repeatedly in the hippocampus of freely behaving rats over a 2.5 month period. The time course of sHFS-induced potentiation from the same animals is displayed before (**B**, 0 month), 2 h after (**C**, 0.5 month), 1 (**D**, 1.5 month) and 2 (**E**, 2.5 month) months after the last injection, starting at 4 months. Red and blue colours indicate CORT 10 or CORT 5 treatments, respectively (online version only). Insets show representative field EPSP traces at the times indicated. Calibration bars: vertical, 1 mV; horizontal, 10 ms. The magnitude of potentiation 3 h post-sHFS (last 10 min) at the different recording sessions is plotted for 10 mg/kg (**F**) and 5 mg/kg (**G**) CORT-treated rats, for individuals (left hand panel) and groups (right hand panel). The ♯ symbol stands for a statistical comparison between pre- and 3 h post-HFS values at each recording session within one group (paired *t* test) whereas an * indicates a comparison between groups. LTP was significantly inhibited at 0.5, 1.5, and 2.5 month in the TG CORT 10 group (one-way ANOVA with repeated measures). In contrast, the magnitude of LTP was not significantly different between recording sessions in the TG CORT 5 group. One symbol, *p* < 0.05; two symbols, *p* < 0.01; three symbols, *p* < 0.001. Values are mean ± S.E.M. % pre-HFS baseline EPSP amplitude.

Next, we examined the ability of sub-chronic administration of the same dose (10 mg/kg/day for 3 days) of CORT to disrupt LTP in freely behaving transgenic rats. We found that this brief CORT treatment caused a very long-lasting inhibition of LTP (Fig. 2). Similar to wild-type rats, LTP was strongly inhibited in transgenic animals at 2 h after the last CORT injection (110 ± 7%, *p* < 0.05 compared with 151 ± 10% of pre-treatment LTP, Fig. 2B, C, F). However, in contrast to wild-type rats, application of sHFS either 1 or 2 months after CORT treatment failed to trigger LTP in these transgenic rats (123 ± 10%, *p* < 0.01 and 102 ± 10%, *p* < 0.001, respectively, compared with pre-treatment LTP, Fig. 2D–F).

To determine if the 10 mg/kg dose was at or near the threshold to cause such a persistent disruption of synaptic plasticity in transgenic rats we also tested half this dose. We found that similar magnitude LTP was triggered at all recording sessions in transgenic rats receiving three injections of 5 mg/kg CORT (Fig. 2).

### Sub-chronic CORT-evoked persistent impairment of NOR performance in APP transgenic rats

**Fig. 3:**
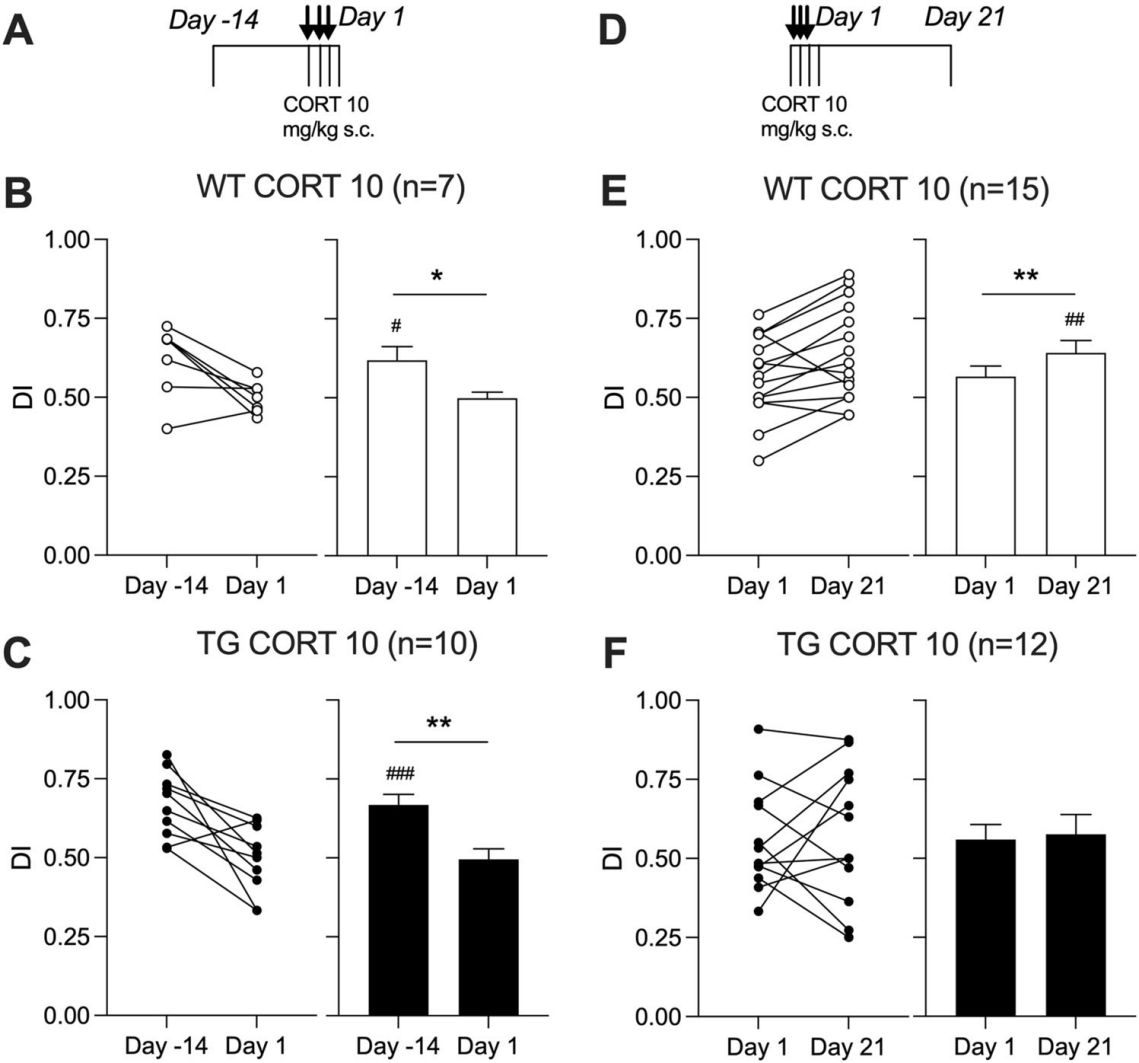
Corticosterone persistently disrupts cognition in APP transgenic rats. In a longitudinal study design (**A**, **D**), wild-type (WT) and transgenic (TG) rats received single daily s.c. injections of CORT (10 mg/kg) for 3 days. In first group of animals, performance of WT (**B**) and TG (**C**) rats was tested in a relatively simple novel object recognition task on Day-14 before CORT treatment and 1 Day after the last CORT injection. To avoid potential confounds of multiple repeated behavioural testing in the NOR task, another group of animals was tested on Days 1 and 21. After initial memory disruption on Day 1, WT animals (**E**) significantly improved DI on Day 21. In contrast, TG rats (**F**) didn’t show any recovery of performance. Summary of discrimination index (DI) for individuals and groups are in left and right hand panels, respectively. The # symbol stands for a statistical comparison of DI with the hypothetical no-bias value of 0.5 on the same day (one sample *t* test) whereas an * indicates a comparison between different days (paired *t* test). One symbol, *p* < 0.05; two symbols, *p* < 0.01; three symbols, *p* < 0.001. Values are mean ± S.E.M. % of DI.

The possibility that the same sub-chronic CORT treatment protocol would also cause a persistent disruption of cognition was next tested using a relatively simple NOR test [42]. First, we confirmed that transgenic rats were not impaired at this pre-plaque age. Both untreated transgenic rats and their wild-type littermates had a similar small but significant bias towards the novel object when tested 3 h after the training session (*p* < 0.001 and *p* < 0.05, respectively, compared with the hypothetical no-bias value of 0.5, one sample *t* test, Supplementary Fig. 2, see also Fig. 3B, D).

In wild-type rats, consistent with the LTP studies (see Fig. 1), sub-chronic CORT reversibly disrupted NOR performance (Fig. 3B). Thus, in these animals on day 1 post-injection there was no preference for the novel object (*p* > 0.05), whereas on day 21 there was a significant bias (*p* < 0.01, Fig. 3E). In contrast, in transgenic rats, and again similar to the LTP studies (see Fig. 2), this sub-chronic CORT regime had a more persistent deleterious effect, impairing performance of the NOR task both on days 1 and 21 (*p* > 0.05 on both days, Fig. 3C, F).

Of note, the performance of the same rats in an open field test 2 h after the last CORT injection, just prior to the NOR task, was statistically indistinguishable. Animals of both genotypes travelled a similar distance (lines crossed, mean ± S.E.M., 90 ± 7 in WT, *n* = 24, vs. 78 ± 7 in TG, *n* = 23; *p* = 0.21) and spent comparable amount of time in the centre (mean ± S.E.M., 14 ± 3 s in WT, *n* = 24 vs. 8 ± 2 s in TG, *n* = 23; *p* = 0.08) (data not shown).

### The glucocorticoid methylprednisolone also persistently inhibits LTP in APP transgenic rats

The disruption of LTP by sub-chronic CORT is likely mediated via glucocorticoid receptor activation [13, 43]. Therefore, we predicted that sub-chronic treatment with another agonist of these receptors, MPL, would be sufficient to mimic this action of CORT. To our surprise, repeated treatment with a relatively high dose (50 mg/kg per day for 3 days) of MPL had no significant effect on sHFS-induced LTP magnitude at any time point in wild-type rats (148 ± 9% for session 0 month and 149 ± 14% for session 2.5 month, Supplementary Fig. 3).

**Fig. 4:**
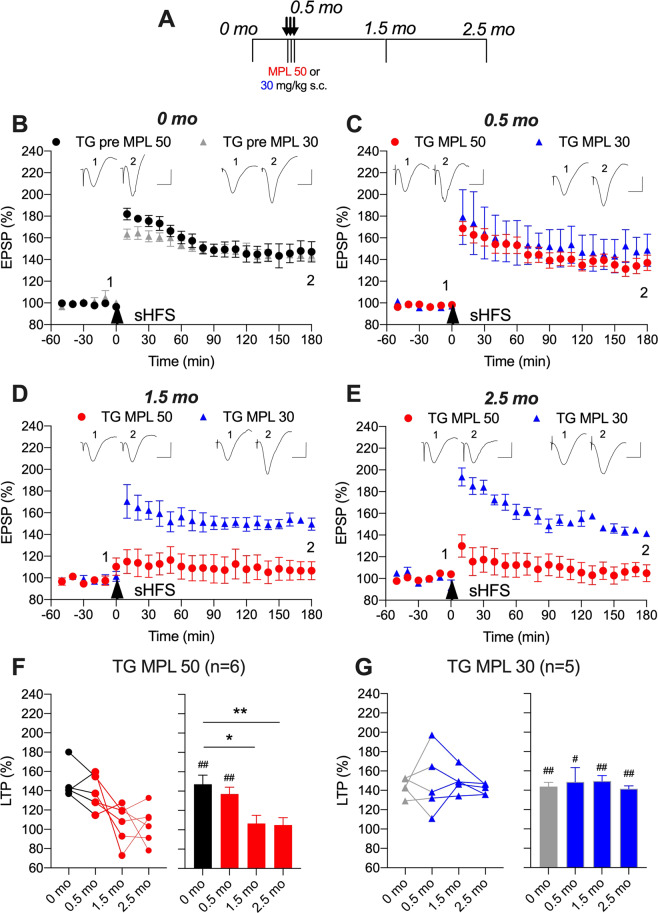
Persistent disruption of synaptic plasticity in APP transgenic rats by the glucocorticoid methylprednisolone. **A** Study design: Transgenic rats (TG) received single daily s.c. injections of methylprednisolone (MPL either 50 mg/kg, closed circles, or 30 mg/kg, open circles) for 3 days. Strong high frequency conditioning stimulation (sHFS, arrow) was applied repeatedly in the hippocampus of freely behaving rats over a 2.5-month period. The time course of sHFS-induced potentiation from the same animals is displayed before (**B**, 0 month), 2 h after (**C**, 0.5 month), 1 (**D**, 1.5 month) and 2 (**E**, 2.5 month) months after the last injection, starting at 5 months. Red and blue colours indicate MPL 50 or MPL 30 treatments, respectively (online version only). Insets show representative field EPSP traces at the times indicated. Calibration bars: vertical, 1 mV; horizontal, 10 ms. The magnitude of potentiation 3 h post-sHFS (last 10 min) at the different recording sessions is plotted for TG MPL 50 (**F**) and TG MPL 30 (**G**) rats, for individuals (left hand panel) and groups (right hand panel). The ♯ symbol stands for a statistical comparison between pre- and 3 h post-HFS values at each recording session within one group (paired *t* test) whereas an * indicates a comparison between groups. The magnitude of LTP was not significantly different between recording sessions in TG MPL 30 group (*p* > 0.05; one-way ANOVA with repeated measures). In contrast, LTP was significantly inhibited at 1.5 and 2.5 month in TG MPL 50 group. One symbol, *p* < 0.05; two symbols, *p* < 0.01. Values are mean ± S.E.M. % pre-HFS baseline EPSP amplitude.

In contrast to wild-type rats, the same MPL treatment protocol had a delayed persistent inhibitory effect in transgenic animals (Fig. 4). Although there was no observable change 2 h after the third injection, LTP was inhibited 1 month later (107 ± 8%, *p* < 0.05 compared with 147 ± 9% of pre-treatment LTP, Fig. 4B, D, F), consistent with a role for glucocorticoid receptors in the prolonged disruptive action of CORT. Importantly, the lower dose of MPL (30 mg/kg) had no effect on LTP in transgenic animals (Fig. 4).

### An NLRP3 inflammasome inhibitor reverses the persistent inhibition of LTP in CORT-treated APP transgenic rats

**Fig. 5:**
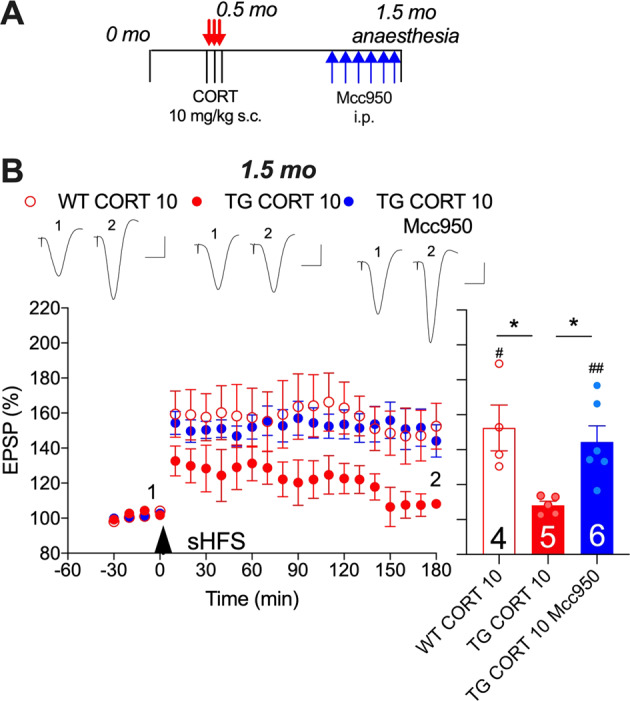
Reversal of the corticosterone-evoked persistent disruption of synaptic plasticity in anaesthetised transgenic rats by the inflammasome inhibitor Mcc950. **A** Schematic diagram of treatment regimen, starting at 5 months of age (online version in colour). **B** Whereas sHFS triggered robust LTP in wild-type rats pretreated 1 month previously with CORT (WT CORT 10), the same pretreatment in transgenic rats strongly inhibited LTP (TG CORT 10). Six-day i.p. treatment with Mcc950 (16 mg/kg/day) reversed the LTP deficit in transgenic rats (TG CORT 10 MCC950). An arrowhead indicates the time point of sHFS application. Inserts show representative EPSP traces at the times indicated. Calibration bars: Vertical, 1 mV, horizontal 10 ms. LTP magnitude was determined during the last 10 min. Statistical summary is in the right hand panel. Number of animals per group is shown on the bar. *p < 0.05 (one-way ANOVA followed by Bonferroni’s multiple comparison test); ^#^*p* < 0. 05, ^##^*p* < 0.01 compared with pre-sHFS baseline (paired *t* test). Values are the mean ± S.E.M. % pre-HFS baseline EPSP amplitude.

Previously we reported that sub-chronic treatment with Mcc950 reversed the disruption of the weaker 200 Hz HFS-induced LTP in pre-plaque transgenic rats [23]. Here, we tested the efficacy of the same treatment protocol in 6-month-old CORT-pretreated transgenic rats to abrogate the persistent impairment of sHFS (400 Hz)-induced LTP. In these experiments we used a cross-sectional study design and recorded LTP under anaesthesia. Consistent with the findings in freely behaving rats (Figs. 1 and 2), 1 month after repeated treatment with CORT the application of sHFS induced robust LTP in wild-type (152 ± 13%) rats but only a weak potentiation in transgenic (108 ± 2%) animals (Fig. 5). Remarkably, i.p. injection with Mcc950 in the last 6 days (16 mg/kg/day) prior to recording reversed the deficit in transgenic rats. Indeed, LTP induced by sHFS in these rats was similar in magnitude to that recorded in CORT-pretreated wild-type animals (144 ± 9%, *p* > 0.05).

Although there was a trend to increased number of Iba1-positive cells in the CA1 area 1 month after the last CORT injection in TG animals (Supplementary Fig. 4 and Supplementary Methods), a more sensitive technique would be needed to detect any CORT-induced, NLRP3-dependent microglia activation.

## Discussion

Recovery from the synaptic plasticity disrupting action of CORT was greatly delayed in an animal model of early Alzheimer’s disease amyloidosis. Whereas in wild-type rats LTP inhibition after brief treatment with CORT was transient, the deficit in LTP lasted greater than 2 month in pre-plaque McGill APP rats. Enduring deficits in NOR memory were also triggered by CORT only in the transgenic animals. Moreover, the glucocorticoid MPL mimicked, and the selective NLRP3 inflammasome inhibitor Mcc950 reversed, the persistent impairment of LTP in the transgenic rats. These findings indicate that early amyloidosis increases the vulnerability of hippocampal synaptic circuits to persistent deleterious actions of glucocorticoids. The inflammasome inhibitor-mediated reversal of this persistent synaptoxicity lends support to further testing this therapeutic strategy in early Alzheimer’s disease.

Corticosteroids and Aß can strongly interfere with similar hippocampal synaptic plasticity mechanisms and consequently have considerable potential to modulate each other’s disruptive effects on LTP [13, 20, 21]. Here, we focused on the endurance of hippocampal synaptic plasticity disruption by corticosteroids both in healthy controls and a well-established transgenic rat model of Alzheimer’s disease amyloidosis. We found that short-term high-dose CORT was sufficient to transiently impair sHFS-induced LTP in wild-type rats. This finding is consistent with previous studies on the acute action of CORT on LTP induced using generally milder conditioning protocols [13, 43]. The latter protocols induce NMDA receptor-dependent LTP whereas sHFS-induced LTP is also voltage-gated Ca^2+^ channel-dependent [26, 28]. Here, short-term CORT acutely impaired a relatively late (3 h) phase of LTP while an earlier phase of LTP (<1 h) appeared unscathed. The apparent lack of change at the earlier phase may indicate that induction mechanisms triggered by the sHFS are relatively resistant to acute CORT.

In vitro, bath-application of CORT has been found to primarily inhibit NMDA receptor-dependent LTP in area CA1 [44–46]. We previously reported that in vivo, both these forms of LTP can be acutely impaired by CORT-elevating acute inescapable stress in wild-type rats [28], but see [44, 47]. Depending on the severity, escapability and duration of the acute stress, recovery of hippocampal NMDA receptor-dependent LTP has been reported to take minutes to over 4 weeks in wild-type animals [28, 48–54]. Although there is a paucity of information available regarding recovery from chronic corticosteroid-mediated inhibition of LTP [12], it is known that hippocampal area CA1 structural synaptic damage caused by high dose long-term CORT exposure reverts to normal between 3 weeks and a month of ceasing treatment in adult wild-type animals [55–57]. Three months injection of CORT in mid-aged (1 year old) wild-type rats caused a deficit in synaptic plasticity that extended to 1 month after ceasing treatment [12]. Thus, our finding that three days of CORT treatment in young pre-plaque transgenic rats impaired LTP for more than 2 months is remarkable. That the disruption of plasticity lasted so long indicates that the disease course was accelerated or aggravated by CORT. It will be important to determine if such brief CORT treatment persistently increases soluble Aß concentration, as has been reported for chronic glucocorticoid exposure in transgenic mice [58]. Recently, prolonged multi-modal ‘modern life-like’ acute stress was reported to rapidly increase Aß oligomers via upregulation of BACE and exacerbate hippocampal CA3 spine loss in transgenic mice [59]. We know that Alzheimer’s disease brain-derived Aß oligomers can strongly inhibit sHFS-induced LTP [60]. In pilot studies we found that sHFS-induced LTP appeared unaffected in 9–10 months old control transgenic rats. Future studies need to investigate how long the CORT-triggered synaptic plasticity disruption lasts and at what age, if any, transgenic rats develop similar deficits in sHFS-induced LTP.

The ability of sub-chronic CORT to persistently inhibit LTP in the transgenic animals was not discernible when half the active dose was given. The requirement for a relatively high dose is consistent with the likely involvement of glucocorticoid receptors. Indeed similar brief administration of the glucocorticoid MPL also strongly inhibited LTP 2 months after the last injection, again in an apparently dose-dependent manner. Somewhat surprisingly, unlike CORT, brief MPL treatment did not significantly impair sHFS-induced LTP when tested 2 h after the last injection in either wild-type or transgenic rats. This difference between the time course of MPL and CORT action may at least partly lie in their different kinetics [61, 62], in particular the rate of penetration into the brain. Unlike CORT [63, 64], MPL has been found to enter the brain slowly by a saturable mechanism [65] and is a good substrate of P-glycoprotein, a major efflux pump of the blood-brain barrier [66]. Consistent with this suggestion the application of sHFS 6 h after the last MPL injection (50 mg/kg, i.p. daily for 3 days) failed to trigger significant LTP in either WT or TG urethane-anesthetized rats (*n* = 4 per group; unpublished observations). Multiple cellular mechanisms have been proposed to mediate the acute glucocorticoid receptor-dependent disruption of synaptic plasticity, including, for example, the Ras–Raf pathway [67] which is also elevated in AD brain [68, 69]. Future experiments should assess the relative roles of these mechanisms in the long-lasting inhibition of LTP by CORT in the TG rats.

Sub-chronic CORT treatment also had more persistent disruptive effects on cognition in transgenic rats as assessed using a relatively simple NOR test. These transgenic rats have been reported not to be impaired on the version used here up to 1 year of age [42], again indicating that the disease course was accelerated or aggravated by CORT. Sub-chronic high-dose MPL is currently used clinically for disorders including certain forms of multiple sclerosis, and has been reported to cause a persistent, but reversible, impairment of memory function in these patients [70, 71]. The possibility of brief glucocorticoid-induced long-lasting deficits in other cognitively vulnerable populations, including early Alzheimer’s disease, needs further research [9, 72].

The NLRP3 inflammasome is a molecular complex central to the production of pro-inflammatory cytokines such as interleukin-1ß [73]. Evidence of its involvement in early Alzheimer’s disease [74] includes enhanced cerebral expression of the key NLRP3 component, active caspase-1 [75]. The NLRP3 inflammasome has been reported to mediate microglial response to either Aß [76] or CORT [24]. Inhibition of the NLRP3 inflammasome reduces Aß burden and enhances cognition in plaque-bearing transgenic mice [75, 77], and reverses the LTP deficit in pre-plaque transgenic rats [23]. The present finding that Mcc950 can also abrogate CORT-exacerbated LTP inhibition supports future evaluation of its efficacy against the CORT-exacerbated behavioural deficits. The finding also strongly supports a requirement for the NLRP3 inflammasome in the persistent deleterious synaptic actions of brief exposure to excess CORT on a background of Alzheimer’s disease amyloidosis.

The present animal model findings on the synaptotoxic interaction between CORT and Aß, taken together with recent prospective longitudinal preclinical Alzheimer’s disease human studies [5, 78], lend support to developing lifestyle changes to mitigate against exposure to glucocorticoid excess and underlie the potential benefit of therapies targeting shared mechanisms such as inflammasome activation early in the disease.

## Funding and disclosure

Research reported here was supported by Science Foundation Ireland (19/FFP/6437 and 14/IA/2571 to M.J.R.), Health Research Board Ireland (ILP-POR-2019-051), National Natural Science Foundation of China (No. 81471114 to N.W.H.) and by Zhengzhou University (140/32310295 to N.W.H.). The authors declare that they have no conflict of interest. Open Access funding provided by the IReL Consortium.

## Supplementary information

Supplementary Table 1

Supplementary Figures, Methods & Table 2
