# Supplementary material for: Enduring glucocorticoid-evoked exacerbation of synaptic plasticity disruption in male rats modelling early Alzheimer’s disease amyloidosis
Source: Neuropsychopharmacology. 2021 Jun 29;46(12):2170–9. doi: 10.1038/s41386-021-01056-9 (PMC8505492; doi:10.1038/s41386-021-01056-9)
Supplement: Supplementary file 2 — Supplementary Figures, Methods & Table 2 [file 41386_2021_1056_MOESM2_ESM.pdf]

Supplementary Information

Enduring glucocorticoid-evoked exacerbation of synaptic plasticity disruption in male rats modelling early Alzheimer's disease amyloidosis

Yingjie Qi[1], Igor Klyubin[1], Tomas Ondrejcak[1], Neng-Wei Hu[1,2] and Michael J. Rowan[1]

[1]Department of Pharmacology &Therapeutics and Institute of Neuroscience, Trinity College, Dublin 2, Ireland; [2]Department of Physiology and Neurobiology, School of Basic Medical Sciences, Zhengzhou University, 100 Science Avenue, Zhengzhou 450001, China

1. **Supplementary Figure 1** Stable induction of long-term potentiation with repeated application of strong conditioning stimulation in chronically implanted McGill-R-Thy1-APP transgenic rats

2. **Supplementary Figure 2** Novel object recognition in naïve 5 month-old wild-type and transgenic McGill rats

3. **Supplementary Figure 3** The selective glucocorticoid methylprednisolone does not inhibit sHFS-induced LTP in wild-type rats

4. **Supplementary Figure 4** Iba1 staining in the brains of transgenic McGill rats

5. **Supplementary Methods** Iba1 and 3A1 staining

6. **Supplementary Table 2.** Summary of statistical comparisons for Supplementary Figures 1- 3

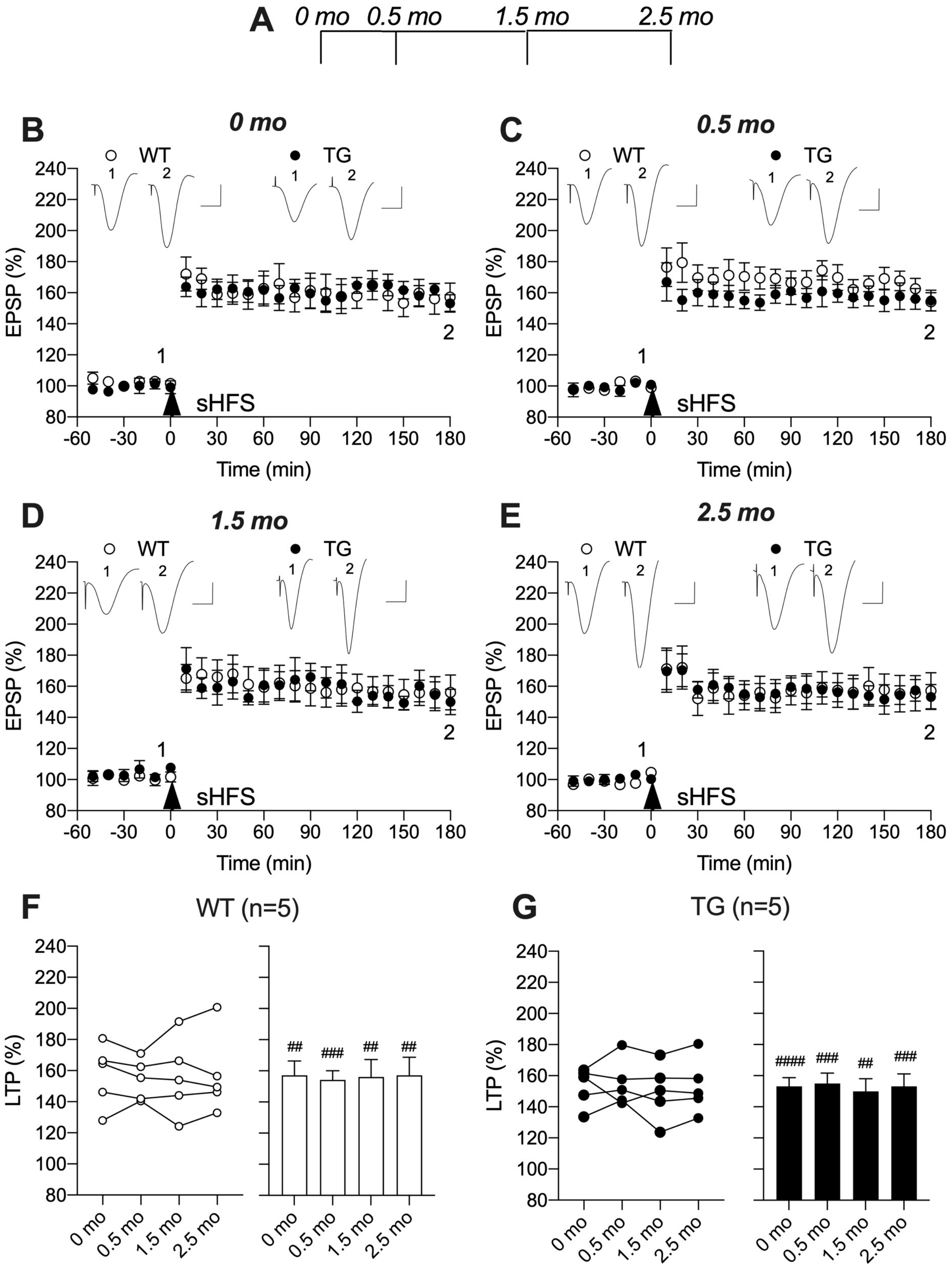

Supplementary Figure 1. Stable induction of long-term potentiation with repeated application of strong conditioning stimulation in chronically implanted McGill-R-Thy1-APP transgenic rats **(A)** In a longitudinal study design, the re-application of strong high frequency stimulation (sHFS; three sets of high intensity 400 Hz trains;

arrowhead) triggered robust LTP in the hippocampus of control (wild-type littermates, WT, open circles) and McGill-R-Thy1-APP transgenic (TG, black circles) freely behaving rats over a 2.5 month period. LTP time course from the same animals is displayed at the 0 (**B**), 0.5 (**C**), 1.5 (**D**) and 2.5 (**E**) month (mo) recording sessions, starting at 4 months of age. Insets show representative field EPSP traces at the times indicated. Calibration bars: vertical, 1 mV; horizontal, 10 ms. The magnitude of potentiation 3 h post-sHFS (last 10 min) at the different recording sessions is plotted for wild-type (**F**) and transgenic (**G**) rats, for individuals (left hand panel) and groups (right hand panel). The # symbol stands for a statistical comparison between pre- and 3 h post- HFS values at each recording session (paired t-test). The magnitude of LTP was not significantly different between recording sessions in either group ($p>0.05$; one-way ANOVA with repeated measures). Two symbols, $p<0.01$; three symbols, $p<0.001$; four symbols, $p<0.0001$. Values are mean±S.E.M. % pre-HFS baseline EPSP amplitude.

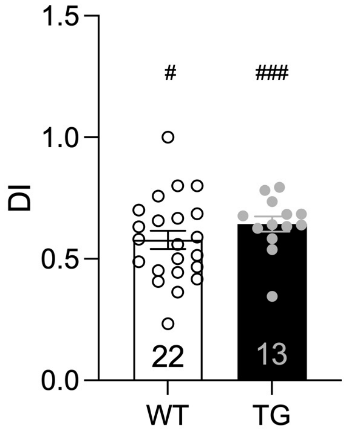

Supplementary Figure 2. Novel object recognition in naïve 5 month-old wild-type and transgenic McGill rats. Both wild-type (WT) and transgenic (TG) animals showed significant learning in a relatively simple task as expressed by discrimination index (DI) in a cross-sectional study. Number of animals per group is shown on the bar. The # symbol stands for a statistical comparison of DI with the hypothetical no-bias value of 0.5 on the same day (one sample t-test) One symbol, $p<0.05$; three symbols, $p<0.001$. Values are mean±S.E.M. % of DI.

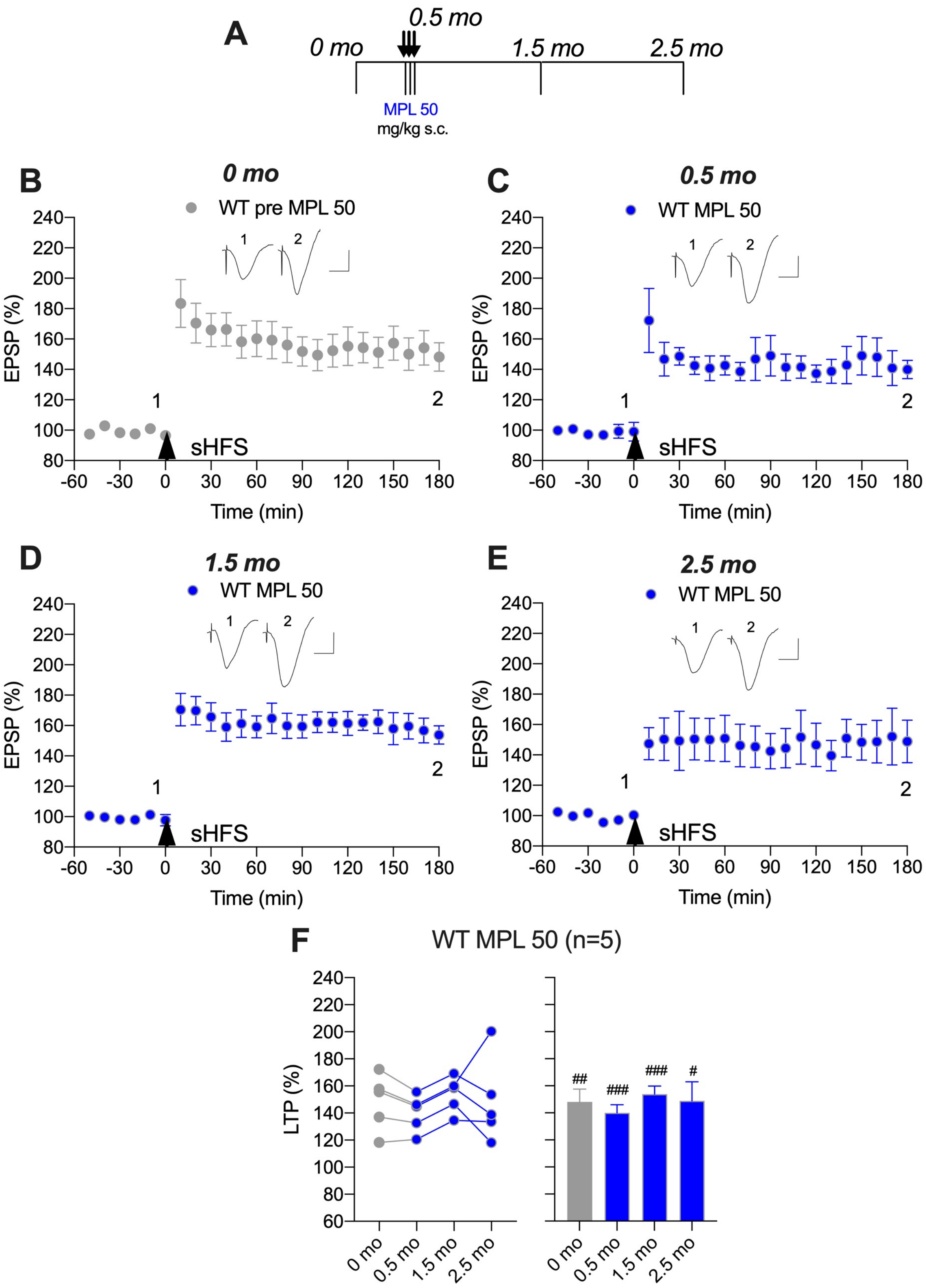

Supplementary Figure 3. The glucocorticoid methylprednisolone does not inhibit sHFS-induced LTP in wild-type rats (**A**) In a longitudinal study design, wild-type (WT) rats received single daily s.c. injections of MPL (50 mg/kg, closed circles) for 3

days. Strong high frequency conditioning stimulation (sHFS, arrowhead) was applied repeatedly in the hippocampus of freely behaving rats over a 2.5 month period. The time course of sHFS-induced potentiation from the same animals is displayed before (**B,** 0 mo), 2 h after (**C,** 0.5 mo), 1 (**D,** 1.5 mo) and 2 (**E,** 2.5 mo) after the last injection, starting at 5 months. Blue colour indicates MPL 50 treatment. Insets show representative field EPSP traces at the times indicated. Calibration bars: vertical, 1 mV; horizontal, 10 ms. The magnitude of potentiation 3 h post-sHFS (last 10 min) at the different recording sessions is plotted in **F** for individuals (left hand panel) and groups (right hand panel). The # symbol stands for a statistical comparison between pre- and 3 h post-HFS values at each recording session within one group (paired t-test). The magnitude of LTP was not significantly different between recording sessions ($p>0.05$; one-way ANOVA with repeated measures). One symbol, $p<0.05$; two symbols, $p<0.01$; three symbols, $p<0.001$. Values are mean±S.E.M. % pre-HFS baseline EPSP amplitude.

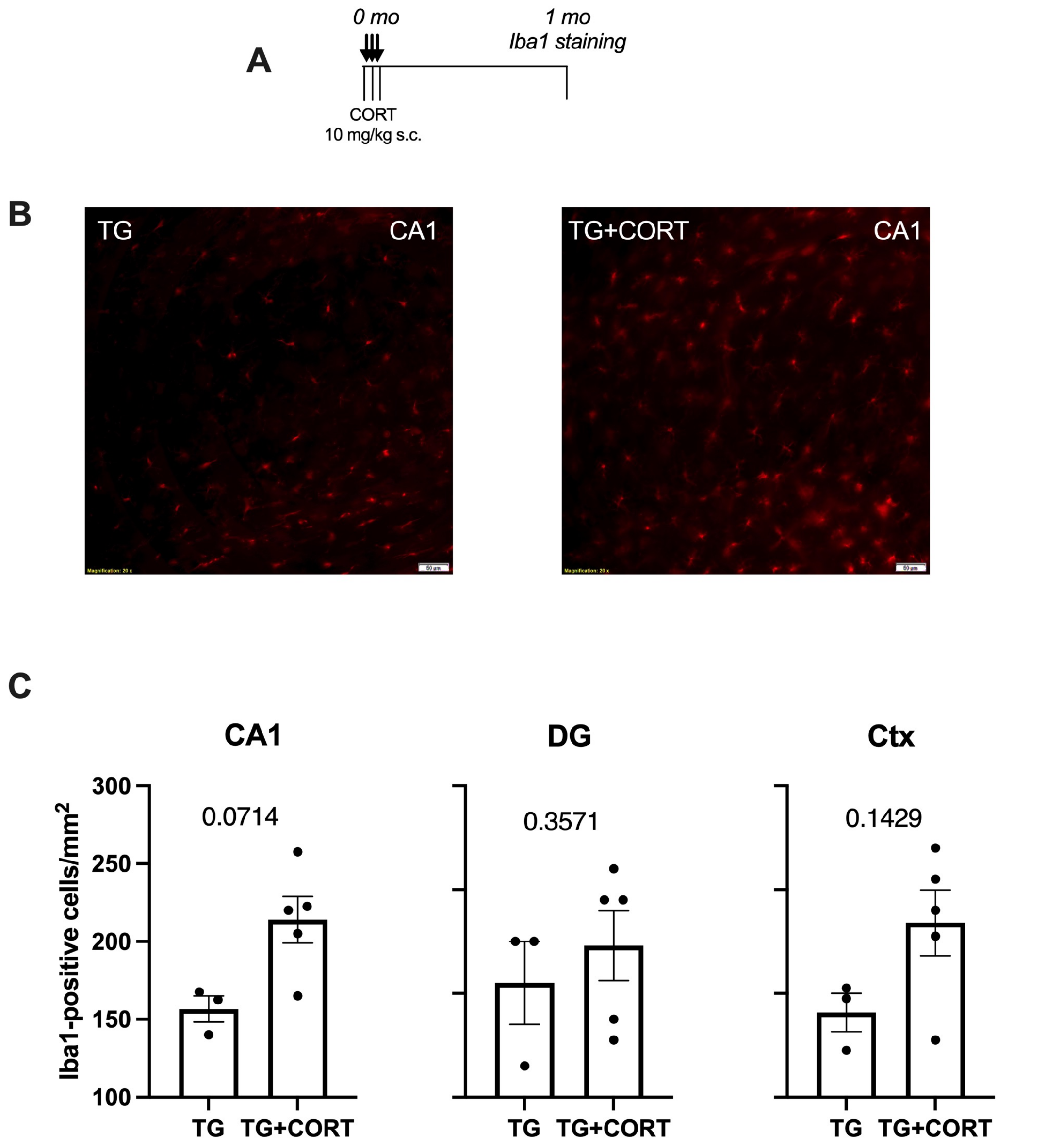

Supplementary Figure 4. Iba1 staining in the brains of transgenic McGill rats. (A) Schematic diagram of treatment regimen. (B) Representative images of the CA1 area of animals treated with CORT (3-day, 10 mg/kg/day, s.c., TG+CORT) or untreated animals (TG). Calibration bar 50 µm. (C) Quantification of the number of microglia as measure by Iba1 staining in different areas of transgenic rat brain. Exact p values (Mann-Whitney U test) are shown above the bars.

Supplementary Methods

Iba1 and 3A1 staining

At the end of electrophysiological experiments the brain was removed under deep urethane anaesthesia and the left (intact) hemisphere was drop-fixed in 4% PFA at 4°C for 24 h. For staining, free-floating coronal hemibrain sections of 70 μm thickness were sliced on a vibrotome (Model 5100mz, Campden Instruments) and incubated overnight in staining solution (4% normal goat serum, 0.4% Triton-X 100 and 4% BSA) with 3A1, an anti-β-amyloid antibody (1:1000, a gift from Dr Brian O'Nuallain, https://www.biolegend.com/en-us/products/purified-anti-beta-amyloid-1-15-antibody-15474) and anti-Iba1 antibody (1:1000, Invitrogen, rabbit, cat.no. PA521274). After washing 3 times with 0.1 M PBS, the free-floating sections were incubated for 1.5 h in staining solution together with anti-mouse Alexa 488 (1:400, Invitrogen, goat, cat.no. A32723) and anti-rabbit Alexa 568 (1:400, Invitrogen, goat, cat.no. A11011). Slices were washed 3 times with PBS and mounted using ProLong Gold mounting medium (Invitrogen, cat.no. P36930). Fluorescence images of brain sections were collected using an epifluorescent microscope (20x objective). The number of cells were mannually counted using ImageJ software. Data were statistically analysed using Mann-Whitney U tests.

We failed to detect any plaques with 3A1 in 6 month-old TG McGill rats, one month after 3-day CORT treatment (10 mg/day/kg s.c., n=5).

Supplementary Table 2. Summary of statistical comparisons for Supplementary Figures 1- 3

Supplementary Figure 1F			
LTP	n	Mean±S.E.M	Baseline vs 3 h post sHFS Paired t-test
WT 0 mo	5	157.16±9.14	p=0.0019, t=7.225, df=4
WT 0.5 mo	5	154.25±5.87	p=0.0001, t=14.32, df=4
WT 1.5 mo	5	156.02±11.24	p=0.0027, t=6.618, df=4
WT 2.5 mo	5	157.13±11.56	p=0.006, t=5.232, df=4
RM one-way ANOVA			
p	0.8447		
F	0.137		
R squared	0.03311		

Supplementary Figure 1G			
LTP	n	Mean±S.E.M	Baseline vs 3 h post sHFS Paired t-test
TG 0	5	153.09±5.62	p<0.0001, t=29.29, df=4
TG 0.5 mo	5	154.95±6.74	p=0.0003, t=11.45, df=4
TG 1.5 mo	5	149.89±8.21	p=0.0034, t=6.207, df=4
TG 2.5 mo	5	153.13±7.96	p=0.0006, t=9.638, df=4
RM one-way ANOVA			
p	0.5859		
F	0.5238		
R squared	0.1158		

Supplementary Figure 2			
Object recognition DI (Discrimination Index)	N	Mean±S.E.M	DI vs hypothetical no-bias index of 0.5 (One sample t-test)
WT	22	0.58±0.04	p=0.0482, t=2.098, df=21
TG	13	0.64±0.03	p=0.0007, t=4.498, df=12
WT vs TG unpaired t-test		Hedges' g= 0.4102575	
p=0.2386, t=1.2, df=33			

Supplementary Figure 3F

LTP	n	Mean±S.E.M	Baseline vs 3 h post sHFS Paired t-test
WT MPL 50, 0	5	148.18±9.34	p=0.0024, t=6.836, df=4
WT MPL 50, 0.5 mo	5	139.00±6.08	p=0.0005, t=10.57, df=4
WT MPL 50, 1.5 mo	5	153.77±5.99	p=0.0001, t=14.97, df=4
WT MPL 50, 2.5 mo	5	148.91±14.04	p=0.04, t=2.998, df=4
RM one-way ANOVA			
p	0.4223		
F	0.8366		
R squared	0.173		
